# Coupling Effects and Analysis in Extremely Large-Scale Planar Array Antennas

**DOI:** 10.3390/s26041109

**Published:** 2026-02-09

**Authors:** Zhiwei Yuan, Zhuxian Lian, Yinjie Su, Yajun Wang, Chuanjin Zu, Bibo Zhang, Lin Ling

**Affiliations:** School of Oceanography, Jiangsu University of Science and Technology, Zhenjiang 212000, China; 19852270080@163.com (Z.Y.);

**Keywords:** extremely large antenna arrays, near-field beamforming, mutual coupling, eigenmode transmission

## Abstract

This paper develops a physically consistent precoding framework for extremely large antenna arrays (ELAAs), incorporating structural mutual coupling through a two-dimensional impedance network. To maintain scalability, we introduce a Neumann series approximation for the inverse coupling operator. Our analysis reveals that coupling-aware received power maximization reduces to a Hermitian rank-one quadratic form, whose optimum aligns with the dominant eigendirection of the effective coupling-shaped channel. This result indicates that both eigen-decomposition-based optimization and coupling-aware maximum ratio transmission (MRT) enhance power efficiency under mutual coupling, with the eigenmode design achieving superior performance. In addition, we further extend the analysis from the free-space path to the multipath scenario, demonstrating the robustness and adaptability of the proposed method under practical propagation conditions. Simulations confirm that structural coupling severely degrades conventional MRT, whereas the proposed eigenmode method with Neumann approximated coupling attains the highest received power among all considered schemes. The framework is interpretable, numerically stable, and readily implementable, offering practical guidance for energy-efficient near-field beamforming on ultra-large apertures.

## 1. Introduction

While the commercial deployment of fifth-generation (5G) mobile networks continues to accelerate worldwide, researchers have already turned their attention to the development of beyond-5G (B5G) and sixth-generation (6G) communication systems. In this context, several advanced transmission technologies have attracted rapidly growing interest, including extremely large-scale multiple-input multiple-output (XL-MIMO) communication [1,2,3], Terahertz (THz) communication [4,5], and intelligent reflecting surface (IRS)-assisted communication [6,7,8,9]. In particular, XL-MIMO extends the paradigm of massive MIMO by scaling antenna arrays to an order of magnitude larger than those in current systems, promising substantial gains in spectral efficiency and network capacity [10,11]. As communication technology evolves toward 6G, antenna scales are further expanding to the level of thousands of elements to support higher data rates, ultra-low latency, and ubiquitous coverage. Thus, extremely large-scale antenna arrays (ELAAs) have emerged as a key enabling technology for 6G communications [12,13,14].

Unlike conventional massive MIMO systems [15,16,17,18], which primarily operate in the far-field region, ELAAs are prone to operating in the near-field region when serving users or scatterers [19,20]. This unique characteristic arises from their significantly extended physical apertures; when combined with the continuous shrinkage of cell sizes in ultra-dense deployment scenarios, this greatly reduces the probability that users or scatterers are located beyond the far-field boundary. The transition from conventional massive MIMO to ELAAs leads to a substantial extension of the Rayleigh distance, a critical boundary that defines the division between the near-field and far-field regions and is mathematically proportional to the square of the array aperture [21,22]. This remarkable expansion of the Rayleigh distance implies that, in numerous practical operational scenarios of ELAA systems, the electromagnetic (EM) radiation field generated by the ELAA shifts from the far-field region to the near-field region. Within the near-field range, the plane-wave assumption, which is widely adopted in traditional channel modeling [23,24,25], no longer holds; instead, spherical-wave effects become predominant. These effects give rise to noticeable phase curvature and non-uniform amplitude variations during signal propagation, which directly alter channel characteristics and introduce spatial non-stationarity [26]. Such changes pose new and unprecedented challenges for accurate channel modeling, efficient beamforming design, and overall system performance optimization. Notably, most existing research works on ELAA systems are still reliant on the plane-wave approximation, thereby overlooking the significant impact of near-field effects on system performance metrics, including ergodic channel capacity. Particularly in the context of the continuous increase in antenna array size—an inevitable trend to meet the ever-growing demand for high data rates and ultra-reliable communication—the accuracy of near-field channel modeling becomes increasingly crucia [27]. It directly determines the effectiveness of subsequent signal processing strategies and the achievable performance upper bound of ELAA systems, making it an indispensable research focus in the development of next-generation communication technologies [26,28].

In XL-MIMO communication systems, antenna elements are typically arranged densely with half-wavelength or smaller spacing, which leads to significant electromagnetic mutual coupling between elements [29,30,31,32]. This structural coupling alters the input impedance distribution at the antenna ports, thereby affecting the current distribution and overall radiation characteristics of the array. Conventional beamforming designs often assume mutually independent antenna elements, neglecting mutual coupling effects, an oversight that can introduce noticeable performance degradation in extremely large arrays [10]. For instance, the authors in [33] investigated the output signal-to-interference-noise ratio (SINR) of an adaptive array while accounting for mutual coupling, with simulation results confirming that coupling degrades array performance. Similarly, ref. [34] examined the impact of mutual coupling in spatially correlated large antenna arrays for millimeter-wave systems, concluding that coupling can significantly reduce spatial correlation for side-by-side dipole elements. In such cases, the change in effective antenna gain caused by mutual coupling becomes a dominating effect and ultimately determines the antenna array performance. Conventional beamforming designs are typically predicated on the assumption of mutually independent antenna elements, neglecting the effects of mutual coupling. This simplification risks overestimating the potential performance gains of ELAAs [10]. Existing research has proposed mutual coupling modeling methods based on impedance matrices. For example, ref. [35] provides an in-depth study of coupling in linear antenna arrays, while studies on mutual coupling in extremely large-scale planar array antennas are relatively scarce. The computational complexity of these methods increases sharply with array size, making them difficult to scale to extremely large arrays, and they lack a joint optimization framework with near-field beamforming.

To address the issues mentioned above, this paper proposes a beamforming framework for extremely large-scale planar arrays that comprehensively considers near-field propagation and structural mutual coupling. The main tasks of this work are: to establish a near-field channel model based on a spherical-wave array response; to characterize inter-element mutual coupling via a two-dimensional impedance network; to introduce a low-complexity matrix inversion method based on the Neumann series approximation; and to obtain the optimal beam through eigen-decomposition. Finally, the performance differences of various beamforming schemes under near-field coupling conditions are compared and analyzed, validating the effectiveness of the proposed framework. This work provides a scalable, physically interpretable theoretical framework and practical guidance for near-field beamforming design in extremely large-scale antenna arrays.

## 2. Materials and Methods

**Figure 1:**
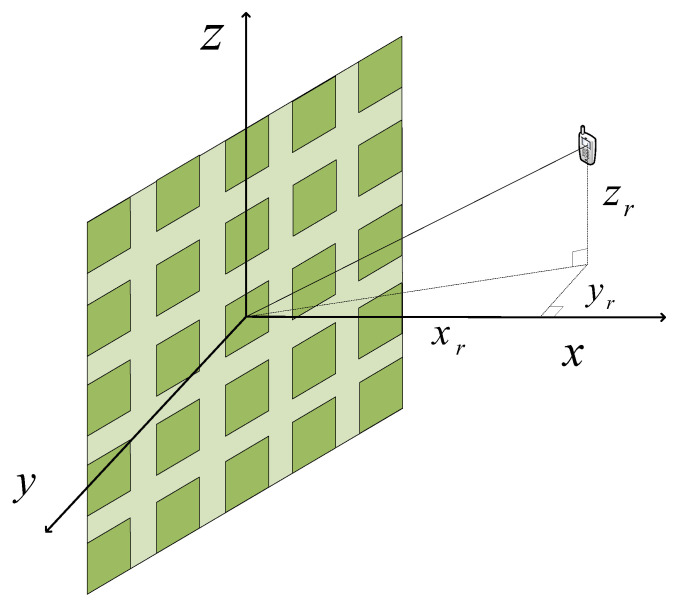
Geometry of the near-field large-scale antenna array link considered in this work.

We consider a large-scale MIMO system in which the transmitter employs a planar antenna array and the receiver uses a single antenna, as illustrated in Figure 1. The transmitter uses an M×N planar array on the YZ-plane (x=0), and the single-antenna receiver is located at $p_{\mathrm{rx}}={\left[x_{r},\;y_{r},\;z_{r}\right]}^{⊤}$. The array comprises *M* rows and *N* columns, for a total of L=MN elements. The carrier wavelength is λ=c/f, where *f* denotes the carrier frequency and *c* is the speed of light. The inter-element spacing is set to half the wavelength, i.e., d=λ/2. The array aperture lies on the yz-plane (thus x=0), and its geometric center is placed at the origin of the three-dimensional Cartesian coordinate system. To maintain the array center exactly at the origin for any array size, we adopt a symmetric indexing scheme for the row position *m* and column position *n*. Specifically, for odd values of *M* and *N*, we use centered integer indices m∈{−(M−1)/2,…,0,…,(M−1)/2} and n∈{−(N−1)/2,…,0,…,(N−1)/2}. For even values of *M* and *N*, the indices are defined as half-integers to ensure symmetry without a central element at the origin: m∈{±1/2,±3/2,…,±(M−1)/2} and n∈{±1/2,±3/2,…,±(N−1)/2}. The physical position of each element (m,n) is consistently given by $p_{m,n}={\left[0,md,nd\right]}^{⊤}$. Under this convention, the Cartesian coordinates of the (m,n)-th element are $p_{m,n}={\left[0,\;md,\;nd\right]}^{T}$; the physical aperture spans (M−1)d and (N−1)d along the *y*- and *z*-axes, respectively, which motivates a near-field treatment for large apertures.

In the large-aperture regime, the far-field plane-wave assumption is no longer valid. We therefore adopt a near-field (spherical-wave) channel model in which propagation is determined by the per-element distance to the receiver at $p_{\mathrm{rx}}={\left[x_{r},\;y_{r},\;z_{r}\right]}^{⊤}$. The distance from the (m,n)-th element to the receiver is given by:

(1) $$r_{m,n}=∥\;p_{\mathrm{rx}}-p_{m,n}\;∥=\sqrt{{\left(x_{r}-0\right)}^{2}+{\left(y_{r}-m\;d\right)}^{2}+{\left(z_{r}-n\;d\right)}^{2}}.$$

Under free-space line-of-sight (LoS) propagation, the complex baseband field of a spherical wave over the path length $r_{m,n}$ exhibits two fundamental effects: (i) a *phase advance* that is linear in the wavenumber k=2π/λ, yielding the factor $e^{-jkr_{m,n}}$, and (ii) an *amplitude decay* due to spherical spreading, for which the field magnitude scales as $1/r_{m,n}$ (and the received power scales as $1/r_{m,n}^{2}$). Ignoring element patterns and polarization, the near-field array-response entry associated with the (m,n)-th element is modeled as [20]:

(2) $$a_{\mathrm{nf}}\left(m,n\right)=\frac{1}{r_{m,n}}\exp\;\left(-j\;\frac{2\pi }{\lambda }\;r_{m,n}\right).$$

where $r_{m,n}$ represents the distance from the (m,n)-th element to the receiver. Unlike the far-field plane-wave approximation, this spherical-wave model accurately captures the phase curvature essential for near-field analysis in ELAAs.

In traditional MIMO systems, it is typically assumed that antenna elements are mutually independent. However, in extremely large-scale antenna arrays, this assumption becomes invalid due to significant mutual coupling effects. Mutual coupling results in electromagnetic interactions between adjacent antenna elements, which in turn alters current distributions and distorts the array radiation pattern, thus degrading overall system performance.

To characterize this effect, we introduce a coupling impedance matrix model. The mutual coupling among elements is represented by a complex impedance matrix $Z_{2D}$, whose entries reflect the self and mutual impedances between antenna elements.

We consider the horizontal and vertical coupling structures of a planar array separately. Let $\Gamma_{h}\in C^{M\times M}$ and $\Gamma_{v}\in C^{N\times N}$ denote the coupling matrices along the horizontal and vertical directions, respectively. Both matrices share the same symmetric tridiagonal Toeplitz structure. We define a generic coupling matrix $\Gamma \left(K\right)\in C^{K\times K}$ with the following structure [35]:

(3) $$\Gamma \left(K\right)=\left[\begin{array}{ccccc} Z_{A} & Z_{M} & \; & & \\ Z_{M} & Z_{A} & Z_{M} & \; & \\ \; & Z_{M} & \ddots & \ddots & \\ \; & \; & \ddots & Z_{A} & Z_{M}\\ \; & \; & & Z_{M} & Z_{A} \end{array}\right],$$

where $\Gamma_{h}=\Gamma \left(M\right)$ and $\Gamma_{v}=\Gamma \left(N\right)$. Here, $Z_{A}$ is the self-impedance of each antenna element, and $Z_{M}$ denotes the mutual impedance between adjacent elements. The mutual impedances are obtained by employing the electromotive force method due to its numerical convenience [36].

The full two-dimensional coupling matrix $\Gamma_{2D}$ is then constructed via the Kronecker product:

(4) $$\Gamma_{2D}=\Gamma_{h}⊗\Gamma_{v}.$$

According to reference [35], the overall coupling matrix that includes the load impedance $Z_{L}$ is given by:

(5) $$Z_{\mathrm{full}}=\Gamma_{2D}+Z_{L}I_{L},$$

where $Z_{L}$ is the load impedance at each antenna port, and $I_{L}\in R^{L\times L}$ denotes the identity matrix of dimension L=MN.

Finally, the complete coupling matrix is given by the formula:

(6) $$Z_{\mathrm{struct}}=\left(Z_{A}+Z_{L}\right){\left(\Gamma_{2D}+Z_{L}I_{L}\right)}^{-1}.$$

Clearly, the ${\left(\Gamma_{2D}+Z_{L}I_{L}\right)}^{-1}$ part of the formula involves considerable computational complexity. Therefore, we use the Neumann series approximation to solve the inverse matrix [28,37]. The detailed derivation process can be found in Appendix A:

(7) $$Z_{\mathrm{approx}}=\left(Z_{A}+Z_{L}\right)\cdot \left(2I-\Lambda^{-1}Z_{\mathrm{full}}\right)\Lambda^{-1}.$$

### 2.1. Eigenmode-Based Beamforming Optimization

#### 2.1.1. Received Complex Baseband Signal

Let $x\in C^{L\times 1}$ denote the transmit beamforming vector (a total power constraint ${∥x∥}_{2}^{2}=P_{t}$ will be imposed in subsequent subsections). After passing through the structural coupling network, the effective port-current (or excitation) becomes Zx, where $Z\in \{I_{L},Z_{\mathrm{struct}},Z_{\mathrm{approx}}\}$ represents, respectively, the no-coupling baseline, the exact impedance-based coupling, or its low-complexity approximation (cf. Section 2). The per-element near-field response toward the receiver is captured by $a_{\mathrm{nf}}\in C^{L\times 1}$ (cf. Section 2), and C>0 denotes a path-loss normalization constant.

Under free-space LoS propagation, the complex baseband received signal can be written as:

(8) $$y=\sqrt{C}\;a_{\mathrm{nf}}^{H}\;Z\;x+n,$$

where *n* models receiver noise (e.g., $n∼\mathrm{CN}(0,\sigma^{2})$). Since the focus of this section is power-maximizing beamforming, we first neglect noise for clarity, which yields:

(9) $$y=\sqrt{C}\;a_{\mathrm{nf}}^{H}\;Z\;x,$$

(10) $$P_{r}={\left|y\right|}^{2}=C\;|a_{\mathrm{nf}}^{H}Zx{|}^{2}.$$

Equation (10) shows that the received power is governed by the coherent projection of the *coupling-shaped* excitation Zx onto the near-field array response $a_{\mathrm{nf}}$, scaled by *C*. This expression forms the objective for the beamforming optimization developed in the remainder of this section.

#### 2.1.2. Uncoupled Baseline: Maximum Ratio Transmission (MRT)

We set $Z=I_{L}$ for the no-coupling baseline. The received complex baseband signal and the corresponding received power are:

(11) $$y=\sqrt{C}\;a_{\mathrm{nf}}^{H}x,$$

(12) $$P_{r}={\left|y\right|}^{2}=C\;|a_{\mathrm{nf}}^{H}x{|}^{2},$$

where $a_{\mathrm{nf}}\in C^{L\times 1}$ denotes the near-field (spherical-wave) array-response vector and $x\in C^{L\times 1}$ is the transmit beamforming vector subject to ${∥x∥}_{2}^{2}=P_{t}$.

Maximizing (12) under the total power constraint is equivalent to:

(13) $$\max_{x\in C^{L}}\;x^{H}Q_{0}x\;s.t.\;{∥x∥}_{2}^{2}=P_{t},\;Q_{0}=a_{\mathrm{nf}}a_{\mathrm{nf}}^{H},$$

where $Q_{0}$ is Hermitian, positive semidefinite, and rank one.

According to the Cauchy–Schwarz inequality, we can deduce that:

(14) $$|a_{\mathrm{nf}}^{H}x|\;\leq \;∥{a_{\mathrm{nf}}∥}_{2}{\;∥x∥}_{2}={∥a_{\mathrm{nf}}∥}_{2}\;\sqrt{P_{t}},$$

with equality if and only if x is collinear with $a_{\mathrm{nf}}$. Hence the MRT beamformer (unique up to a global phase) is:

(15) $$x_{\mathrm{MRT}}^{★}=\sqrt{P_{t}}\;\frac{a_{\mathrm{nf}}}{∥a_{\mathrm{nf}}{∥}_{2}},$$

and the optimal received power is:

(16) $$P_{r}^{★}\left(\mathrm{MRT}\right)=C\;P_{t}\;{∥a_{\mathrm{nf}}∥}_{2}^{2}.$$

#### 2.1.3. Eigen-Decomposition Based Beamforming (EVD)

Based on the received signal and power in (10), we rewrite the power using ${\left|u\right|}^{2}=u^{*}u$ as a Hermitian quadratic form.

Define the Hermitian matrix:

(17) $$Q=\left(Z^{H}a_{\mathrm{nf}}\right){\left(Z^{H}a_{\mathrm{nf}}\right)}^{H}=Z^{H}\;a_{\mathrm{nf}}\;a_{\mathrm{nf}}^{H}\;Z,$$

so that the received power can be written as a quadratic form $P_{r}=C\;x^{H}Qx$. Maximizing $P_{r}$ under the total power constraint is therefore equivalent to the Rayleigh-quotient program:

(18) $$\max_{x\in C^{L}}\;x^{H}Qx\;s.t.\;{∥x∥}_{2}^{2}=P_{t}.$$

Let the effective channel be $h_{\mathrm{eff}}=Z^{H}a_{\mathrm{nf}}\in C^{L\times 1}$. Then Q admits the rank–one outer-product representation:

(19) $$Q=h_{\mathrm{eff}}h_{\mathrm{eff}}^{H}.$$

*(i) Hermitian.* Using ${\left(\mathrm{AB}\right)}^{H}=B^{H}A^{H}$ and ${\left(\cdot \right)}^{HH}=\left(\cdot \right)$:

$$Q^{H}={\left(h_{\mathrm{eff}}h_{\mathrm{eff}}^{H}\right)}^{H}=h_{\mathrm{eff}}h_{\mathrm{eff}}^{H}=Q.$$

*(ii) Positive semidefinite.* For any $v\in C^{L}$:

(20) $$v^{H}Qv=v^{H}h_{\mathrm{eff}}h_{\mathrm{eff}}^{H}v={\left(h_{\mathrm{eff}}^{H}v\right)}^{*}\left(h_{\mathrm{eff}}^{H}v\right)=|h_{\mathrm{eff}}^{H}v{|}^{2}\geq 0.$$

Thus Q⪰0.

*(iii) Rank one.* If $h_{\mathrm{eff}}=0$, then Q=0 and rank(Q)=0. Otherwise, for any x, $Qx=h_{\mathrm{eff}}\left(h_{\mathrm{eff}}^{H}x\right)$, which is always a scalar multiple of $h_{\mathrm{eff}}$. Therefore $\mathrm{Range}\left(Q\right)=\mathrm{span}\left\{h_{\mathrm{eff}}\right\}$ is one-dimensional and rank(Q)=1. Equivalently, $Qh_{\mathrm{eff}}={∥h_{\mathrm{eff}}∥}_{2}^{2}\;h_{\mathrm{eff}}$ shows that $h_{\mathrm{eff}}$ is an eigenvector with the unique nonzero eigenvalue $\lambda_{1}={∥h_{\mathrm{eff}}∥}_{2}^{2}$, while all vectors orthogonal to $h_{\mathrm{eff}}$ lie in the null space of Q.

Since Q is Hermitian rank one, its eigen-decomposition is [38]:

(21) $$Q=U\;\Lambda \;U^{H},\;\Lambda =\mathrm{diag}\left(\lambda_{1},0,\ldots ,0\right),\;\lambda_{1}={∥h_{\mathrm{eff}}∥}_{2}^{2},$$

with the (unit–norm) dominant eigenvector:

(22) $$u_{1}=\frac{h_{\mathrm{eff}}}{∥h_{\mathrm{eff}}{∥}_{2}}.$$

The optimizer of (18) is the dominant eigenvector scaled to meet the power constraint:

(23) $$x_{\mathrm{EVD}}^{★}=\sqrt{P_{t}}\;u_{1}=\sqrt{P_{t}}\;\frac{Z^{H}a_{\mathrm{nf}}}{∥Z^{H}a_{\mathrm{nf}}{∥}_{2}}.$$

The EVD solution thus coincides with coupling-aware MRT: normalize the effective channel $h_{\mathrm{eff}}=Z^{H}a_{\mathrm{nf}}$ and scale by $\sqrt{P_{t}}$. In practice, one need not explicitly form or factorize Q; computing and normalizing $h_{\mathrm{eff}}$ avoids large-scale EVD and improves numerical stability.

Then the received power under the coupling-aware eigenmode precoding is given by:

(24) $$P_{\mathrm{eig}}={\left|a^{H}Z_{\mathrm{full}}^{-1}w\right|}^{2}.$$

By substituting the optimal precoding vector $w_{\mathrm{opt}}$, which is the principal eigenvector of the matrix $Q={\left(a^{H}Z_{\mathrm{full}}^{-1}\right)}^{H}\left(a^{H}Z_{\mathrm{full}}^{-1}\right)$, the maximum received power can be expressed as:

(25) $$P_{\mathrm{opt}}=\max_{∥w∥=1}w^{H}Qw=\lambda_{\max}\left(Q\right),$$

where $\lambda_{\max}\left(Q\right)$ represents the maximum available power gain achievable by the proposed eigenmode method.

### 2.2. Channel Model with Multipath Scattering

To evaluate the robustness of the proposed near-field coupling optimization in realistic electromagnetic environments, we extend the deterministic line-of-sight (LoS) model to a comprehensive multipath channel model. Following the statistical framework for millimeter-wave propagation in [39], the aggregate channel vector $a\in C^{L\times 1}$ is characterized by the superposition of a dominant LoS path and multiple non-line-of-sight (NLoS) scattering components [39],

(26) $$a=a_{\mathrm{LoS}}+\sum_{p=1}^{P}a_{\mathrm{NLoS},p},$$

where *P* denotes the number of resolvable scattering clusters caused by environmental obstacles.

#### 2.2.1. Deterministic LoS Component

The LoS component $a_{\mathrm{LoS}}$ represents the direct spherical wavefront from the transmitter to the receiver in the near-field region. The *l*-th element is:

(27) $$a_{\mathrm{LoS},l}=\sqrt{\frac{K}{K+1}}\frac{1}{r_{0,l}}e^{-j\frac{2\pi }{\lambda }r_{0,l}},$$

where *K* is the Rician factor representing the power ratio between LoS and NLoS components, and $r_{0,l}=∥p_{l}-q_{0}∥$ is the distance from the *l*-th antenna element at position $p_{l}$ to the intended receiver at position $q_{0}$.

#### 2.2.2. Stochastic NLoS Component with Uniform Spherical Wavefront

In near-field scenarios, all multipath components exhibit spherical wavefronts at the receiver array. Each NLoS path corresponds to a scattering cluster with distinct spatial characteristics. The *l*-th element of the *p*-th NLoS component is modeled as:

(28) $$a_{\mathrm{NLoS},p,l}=\sqrt{\frac{\beta_{p}}{K+1}}\alpha_{p}\frac{1}{r_{p,l}}e^{-j\frac{2\pi }{\lambda }r_{p,l}},$$

where:

- $\alpha_{p}∼\mathrm{CN}\left(0,1\right)$ is the complex gain of the *p*-th scattering cluster, capturing both amplitude fluctuation and random phase shift;
- $r_{p,l}=∥p_{l}-q_{p}∥$ is the distance from the *l*-th antenna element to the *p*-th scatterer at position $q_{p}$;
- $\beta_{p}$ is the power fraction of the *p*-th NLoS path, satisfying $\sum_{p=1}^{P}\beta_{p}=1$.

The total NLoS power is normalized such that $E\left[∥a_{\mathrm{NLoS}}{∥}^{2}\right]=\frac{1}{K+1}$, ensuring that the total channel power $E\left[{∥a∥}^{2}\right]=1$ when considering unit antenna gains.

## 3. Results

This section presents simulation results that evaluate the impact of structural mutual coupling and different beamforming strategies in a near-field large-scale MIMO system. Unless otherwise stated, the planar antenna array consists of M=101 rows and N=101 columns, with an inter-element spacing of d=λ/2 and a carrier frequency of 30 GHz. The receiver is positioned at $p_{\mathrm{rx}}=\left[0.3,-0.2,0.5\right]$ m. The self-impedance of each antenna element is set to $Z_{A}=73+42.5j\;\Omega$, and the mutual impedance between adjacent elements is $Z_{M}=5-5j\;\Omega$. According to the theoretical derivation of the approximation method, the Neumann series expansion becomes invalid when the coupling matrix loses its diagonal dominance. For comparative analysis, both $Z_{A}$ and $Z_{M}$ are also assigned the same value of 73+42.5jΩ in a separate evaluation, following the parameter values established in [34].

Five transmission schemes are compared: (i) uncoupled maximum ratio transmission (MRT), (ii) MRT with structural mutual coupling, (iii) uniform excitation under coupling, (iv) MRT based on the Neumann series coupling approximation, and (v) eigenmode-based optimization using the approximate coupling matrix. The received power is evaluated over transmit power levels ranging from 10 dBm to 40 dBm.

**Figure 2:**
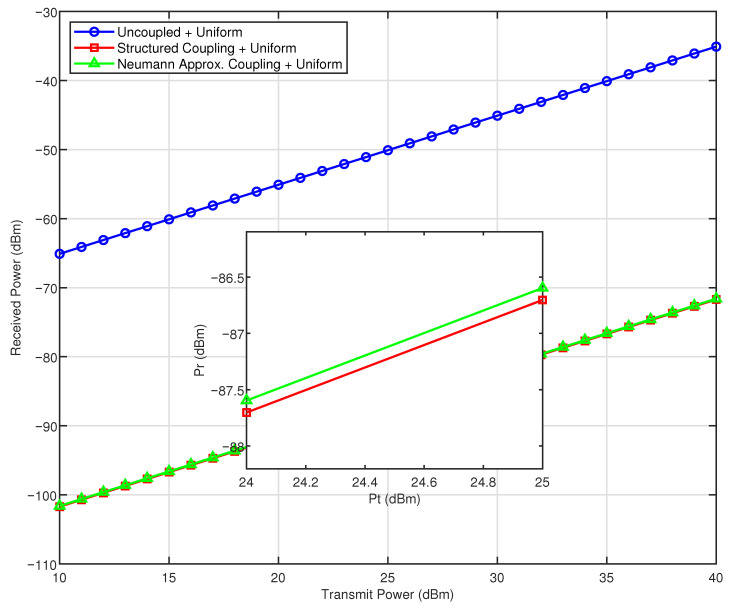
The impact of structured coupling and approximate coupling on the received signal power.

Figure 2 shows the effect of structural and approximate coupling on the received signal power. It compares the performance of three transmission schemes: uncoupled MRT (theoretical upper bound), MRT with full structural coupling, and MRT based on the Neumann series approximation. In the uncoupled case, received power increases linearly with transmit power, representing ideal transmission without mutual coupling. Introducing structural coupling causes a significant drop in received power, highlighting the performance degradation due to mutual coupling in dense arrays. Although the Neumann series approximation does not completely remove coupling effects, it provides a computationally efficient approximation and leads to a modest performance improvement.

**Figure 3:**
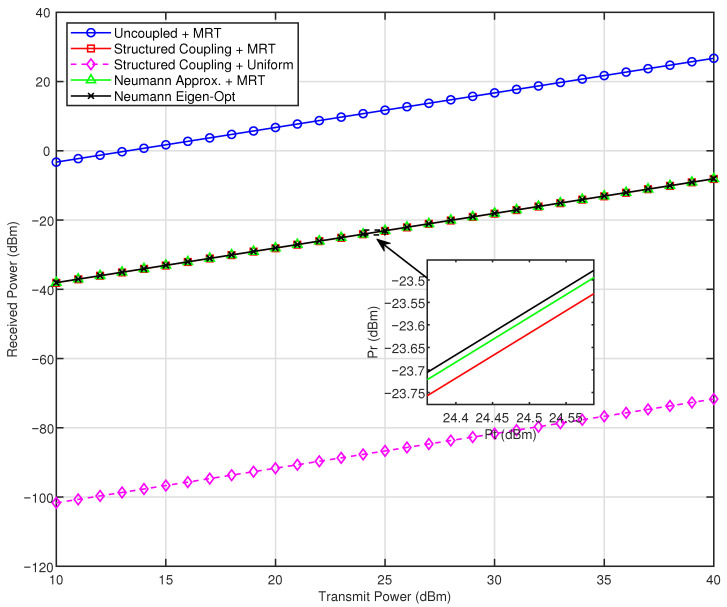
The impact of various optimized coupling schemes on the received signal power.

Figure 3 further examines how different optimized coupling schemes affect the received signal power measured at test points. The curves show that once optimization is applied, both the full structured-coupling model and its Neumann series approximation deliver clear performance gains. Using the full structured-coupling matrix as the optimization variable gives a strong boost in received power, proving that properly handling mutual coupling can effectively offset its usual performance degradation. On the other hand, using the Neumann approximation for matrix inversion achieves nearly the same improvement while significantly reducing computational load. Moreover, the eigenmode transmission method slightly outperforms the coupling-aware MRT in numerical results.

**Figure 4:**
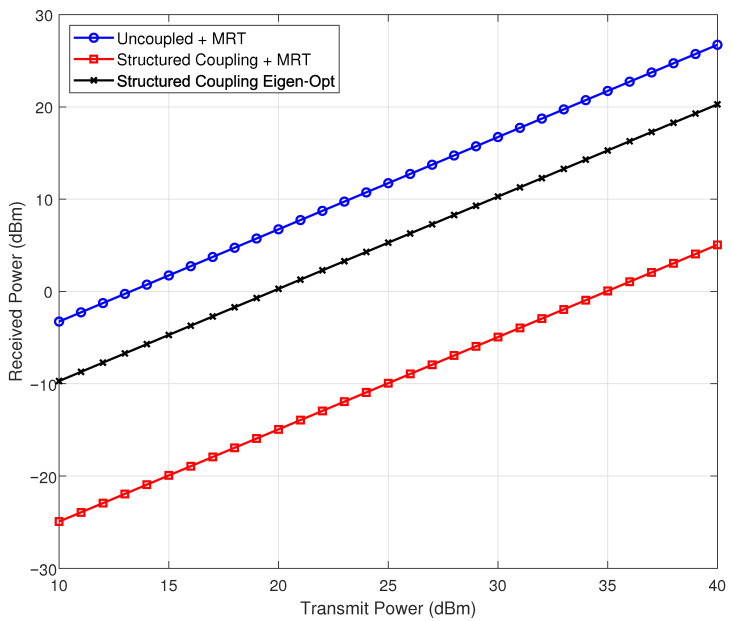
Received signal power comparison when Neumann approximation is inapplicable.

In Figure 4, the mutual impedance $Z_{M}$ between adjacent elements is modified. Under these conditions, the coupling matrix loses its diagonal dominance, rendering the Neumann series approximation inapplicable. Consequently, eigenvalue decomposition is performed directly on the structured coupling matrix. The results show that eigenvector-based optimization provides a measurable improvement in received signal power over conventional approaches.

**Figure 5:**
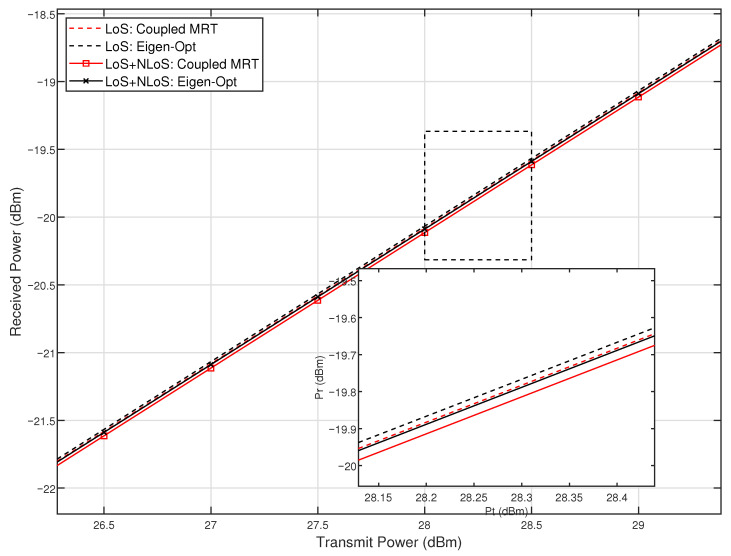
Comparison of received power performance of different beamforming schemes under two channel conditions: line-of-sight (LoS) and multipath (LoS + NLoS, K = 10 dB).

Figure 5 further comparatively analyzes the received power performance of different beamforming schemes in a near-field multipath propagation environment. The simulation curves show that, under line-of-sight (LoS) propagation conditions, the eigen-optimization-based beamforming scheme exhibits a significant performance advantage compared to the maximum ratio transmission (MRT) scheme based on the structural coupling model. This result verifies the effectiveness of the eigen-optimization method as predicted by the theoretical analysis. When non-line-of-sight (NLoS) multipath scattering components are introduced into the channel, the received power of all schemes decreases to a certain extent, reflecting the general challenge posed by scattering environments to beamforming performance. However, under multipath conditions, the eigen-optimization scheme still maintains a performance advantage relative to the structural coupling MRT scheme, demonstrating its relatively good robustness in complex channel environments. The locally enlarged view further indicates that the eigen-optimization method can maintain stable performance gains across different transmit power levels. In summary, the eigen-optimization method not only effectively suppresses mutual coupling effects between antennas but also exhibits favorable transmission performance under the influence of multipath interference, thereby providing a reliable technical approach for beamforming design in practical near-field communication systems.

## 4. Discussion

The simulation results clearly demonstrate the detrimental impact of mutual coupling on near-field beamforming performance in extremely large-scale planar arrays. The proposed eigenmode-based optimization, which leverages the Hermitian structure of the coupling-adjusted channel, achieves significant performance gains over conventional MRT under strong coupling conditions. The Neumann series approximation provides a computationally efficient alternative to exact coupling inversion while maintaining competitive performance.

The equivalence between eigen-decomposition-based optimization and coupling-aware MRT brings clear implementation perks, since it sidesteps the heavy eigen-decomposition step and instead leans on straightforward channel normalization. This insight turns out to be especially handy for real-time beamforming when the antenna count scales to ultra-large arrays.

## 5. Conclusions

This paper mainly introduces a complex large-scale planar array antenna considering structural coupling. The channel is modeled by the spherical wave array response, and the mutual coupling is derived in the form of a Kronecker product. In order to maintain the scalability, the Neumann series approximation of inverse coupling operator is proposed, which avoids the huge amount of calculation of direct matrix inversion.

On this basis, we demonstrate that coupling-aware power maximization exhibits a rank-one Hermitian structure and that the eigen-decomposition-based solution is equivalent to coupling-aware maximum ratio transmission (MRT). This leads to a simple and numerically stable implementation—achieved by normalizing the effective channel instead of performing large-scale eigen-decomposition. The simulation results confirm the theoretical analysis: uncoupled MRT serves as an ideal performance upper bound. Among coupling-aware designs, eigen-decomposition with Neumann approximation yields the highest received signal power, followed by MRT based on the Neumann model, whereas non-adaptive MRT with full structural coupling and uniform excitation exhibits poorer performance. These findings highlight the necessity of incorporating mutual coupling into near-field beamforming design, particularly for systems with large apertures.

In addition to the baseline LoS analysis, we extend our investigation to multipath-rich environments to evaluate the robustness of the proposed framework. Simulation results in Rician fading channels (K = 10 dB) reveal that while all schemes experience performance degradation due to scattering-induced interference, the eigen-optimization approach maintains its superiority. Specifically, it demonstrates improvement over conventional coupling-aware MRT in multipath scenarios, effectively mitigating both mutual coupling and inter-path interference. Furthermore, the performance gap between eigen-optimization and the ideal uncoupled upper bound widens compared to pure LoS conditions, highlighting the compounded challenges of near-field operation in scattering environments. These findings underscore the necessity of joint optimization that simultaneously addresses mutual coupling and multipath characteristics, particularly for large-scale arrays deployed in practical propagation conditions. Future research will integrate element radiation patterns and polarization effects, extend the framework to wideband and multi-user scenarios, and further investigate calibration and hardware non-idealities.

## Data Availability

The original contributions presented in this study are included in the article. Further inquiries can be directed to the corresponding author.

## Appendix A

In this section, we derive the Neumann series approximation used for inverting the full coupling matrix $Z_{\mathrm{full}}$. This is very important to establish the mutual coupling effect model of a super large antenna array.

The impedance matrix $Z_{\mathrm{full}}$ that characterizes the structural mutual coupling in the antenna array is expressed as:

$$Z_{\mathrm{full}}=\Gamma_{2D}+Z_{L}I,$$

where $\Gamma_{2D}$ represents the full 2D coupling matrix, formed by the Kronecker product of the horizontal and vertical 1D coupling matrices:

$$\Gamma_{2D}=\Gamma_{h}⊗\Gamma_{v}.$$

Here, $\Gamma_{h}$ and $\Gamma_{v}$ are the horizontal and vertical coupling matrices, which are based on the self-impedance $Z_{A}$ and mutual impedance $Z_{M}$ between antenna elements.

Assuming that $|Z_{A}\left|\;\gg \;\right|Z_{M}|$, which is typically the case when the antenna elements are densely arranged but well designed, the matrix $Z_{\mathrm{full}}$ is diagonally dominant. Consequently, its inverse can be approximated using the Neumann series [37]. We decompose $Z_{\mathrm{full}}$ into a diagonal part and a perturbation term:

$$Z_{\mathrm{full}}=\Lambda +R,$$

where $\Lambda =\mathrm{diag}(Z_{\mathrm{full}})$ is the diagonal part containing the self-impedances, and $R=Z_{\mathrm{full}}-\Lambda$ is the perturbation term representing the mutual coupling effects. The matrix Λ is invertible, as all diagonal elements are non-zero.

Define the normalized residual matrix T as:

$$T=\Lambda^{-1}R=\Lambda^{-1}\left(Z_{\mathrm{full}}-\Lambda \right).$$

Thus, the full matrix $Z_{\mathrm{full}}$ can be rewritten as:

$$Z_{\mathrm{full}}=\Lambda \left(I+T\right).$$

Taking the inverse of both sides, we obtain:

$$Z_{\mathrm{full}}^{-1}={\left(I+T\right)}^{-1}\Lambda^{-1}.$$

When the spectral radius ρ(T)<1, the inverse ${\left(I+T\right)}^{-1}$ can be expanded using the Neumann series:

$${\left(I+T\right)}^{-1}=\sum_{k=0}^{\infty }{\left(-T\right)}^{k}.$$

In the derivation process, we need to truncate the sequence after two terms in order to calculate the portability:

$${\left(I+T\right)}^{-1}\approx I-T.$$

Thus, the inverse of $Z_{\mathrm{full}}$ is approximated as:

$$Z_{\mathrm{full}}^{-1}\approx \left(I-T\right)\Lambda^{-1}.$$

Compared with the direct inverse matrix, this approximation significantly reduces the computational cost $Z_{\mathrm{full}}$. To construct the approximate coupling matrix $Z_{\mathrm{approx}}$ for beamforming, we substitute the inverse into the coupling model:

$$Z_{\mathrm{approx}}=\left(Z_{A}+Z_{L}\right)\cdot \left(2I-\Lambda^{-1}Z_{\mathrm{full}}\right)\Lambda^{-1}.$$

This approximation avoids the costly matrix inversion while preserving the physical coupling effects with low computational overhead. The Neumann series converges if:

∥T∥ <1orρ(T)<1,

which is generally satisfied due to the relatively small value of $Z_{M}$ compared to $Z_{A}$. Therefore, this approximation is both valid and efficient for large-scale antenna arrays where coupling effects are modest.
